# Acrolein- and 4-Aminobiphenyl-DNA adducts in human bladder mucosa and tumor tissue and their mutagenicity in human urothelial cells

**DOI:** 10.18632/oncotarget.1954

**Published:** 2014-05-07

**Authors:** Hyun-Wook Lee, Hsiang-Tsui Wang, Mao-wen Weng, Yu Hu, Wei-sheng Chen, David Chou, Yan Liu, Nicholas Donin, William C. Huang, Herbert Lepor, Xue-Ru Wu, Hailin Wang, Frederick A. Beland, Moon-shong Tang

**Affiliations:** ^1^ Department of Environmental Medicine, New York University School of Medicine, Tuxedo Park, New York; ^2^ Department of Urology, New York University School of Medicine, New York, New York; ^3^ The State Key Laboratory of Environmental Chemistry and Ecotoxicology, Research Center for Eco-Environmental Sciences, Chinese Academy of Sciences, Beijing 100085, China; ^4^ Division of Biochemical Toxicology, National Center for Toxicological Research, Jefferson, AR

**Keywords:** Bladder cancer, 4-aminobiphenyl, Acrolein, DNA repair, Mutagenesis

## Abstract

Tobacco smoke (TS) is a major cause of human bladder cancer (BC). Two components in TS, 4-aminobiphenyl (4-ABP) and acrolein, which also are environmental contaminants, can cause bladder tumor in rat models. Their role in TS related BC has not been forthcoming. To establish the relationship between acrolein and 4-ABP exposure and BC, we analyzed acrolein-deoxyguanosine (dG) and 4-ABP-DNA adducts in normal human urothelial mucosa (NHUM) and bladder tumor tissues (BTT), and measured their mutagenicity in human urothelial cells. We found that the acrolein-dG levels in NHUM and BTT are 10-30 fold higher than 4-ABP-DNA adduct levels and that the acrolein-dG levels in BTT are 2 fold higher than in NHUM. Both acrolein-dG and 4-ABP-DNA adducts are mutagenic; however, the former are 5 fold more mutagenic than the latter. These two types of DNA adducts induce different mutational signatures and spectra. We found that acrolein inhibits nucleotide excision and base excision repair and induces repair protein degradation in urothelial cells. Since acrolein is abundant in TS, inhaled acrolein is excreted into urine and accumulates in the bladder and because acrolein inhibits DNA repair and acrolein-dG DNA adducts are mutagenic, we propose that acrolein is a major bladder carcinogen in TS.

## INTRODUCTION

Bladder cancer is the fifth most frequently found tumor in the United States, with >70,000 incident cases and resulting in >15,000 deaths annually [1, 2]. Tobacco smoke is the major cause of bladder cancer in United States and smokers are approximately 5 times more likely to get bladder cancer than nonsmokers [3]. Tobacco smoke contains more than 60 carcinogens, however, which carcinogens are the major causes for bladder cancer is not well established [4-6].

It is well known that the incidence of bladder cancer in individuals working in the printing, dyeing, and tanning industries is higher than age- and sex- matched controls [5, 7]. Ample evidence from both epidemiological studies and animal models has firmly established that arylamines, particularly, 4-aminobiphenyl (4-ABP), are the major culprits in bladder cancer related to occupational exposures [8, 9]. Moreover, we have found that in cultured human bladder cells, 4-ABP-DNA adducts are preferentially formed at p53 mutational hotspots (codons 280 and 285) in 4-ABP-related bladder cancer [10, 11]. These results establish a molecular link between carcinogen-induced DNA damage and the p53 mutational patterns in bladder cancer.

The p53 mutational patterns in tobacco smoke-related bladder cancer and in non-smoking-related bladder cancer show distinct differences (Supplementary Figure S1) [12]. While codons 280 and 285 are mutational hotspots in both smokers and non-smokers, codon 273 is a mutational hotspot in tobacco smoke-related bladder cancer but not in non-smoking-related bladder cancer [12]. It has been observed that the formation of 4-ABP-DNA adducts at codon 273 of the p53 gene in urothelial cells treated with electrophilic 4-ABP metabolites is negligible [10, 11]. Acrolein, in contrast, binds preferentially at codon 273 [13, 14]. These results raise the possibility of agents in tobacco smoke other than 4-ABP may cause mutations at codon 273 in bladder cancer.

Because of the strong epidemiological and biochemical evidence, it is generally assumed that 4-ABP is the major bladder carcinogen in tobacco smoke [8, 9]. This assumption has a couple of inherent problems. First, the amount of arylamines in tobacco smoke is minute (1-6 ng/cigarette) [6], and the levels of 4-ABP-DNA adducts in bladder tissue of tobacco smokers are extremely low [15, 16]. Whether or not these low levels of 4-ABP-DNA adduct are responsible for the majority of bladder carcinogenesis events is worthy of careful scrutiny. Second, in addition to arylamines, tobacco smoke also contains acrolein (150-220 μg/cigarette) [6], which has been suggested to cause human bladder cancer [17-20]. Acrolein also has been demonstrated to cause bladder tumor in rat models in combination with uracil [18]. Although acrolein bladder carcinogenicity has not been established humans [21, 22], evidence from prior investigations has suggested that acrolein is a major human lung cancer etiological agent in tobacco smoke [13, 23], thus, we believe an investigation into the role of acrolein in bladder cancer can potentially lead to a better understanding of the pathogenesis of tobacco smoke-related bladder cancer, as well as impact risk assessments and the design of effective prevention measures for tobacco smoke-related bladder cancer.

The level of acrolein in tobacco smoke is 50,000-fold higher than the level of arylamines [6, 15, 24]. Previously, we have found that acrolein inhibits DNA repair and forms acrolein-deoxyguanosine (acrolein-dG) DNA adducts that are mutagenic [25, 26]. Acrolein DNA adducts preferentially form at -CpG- sites, including codon 273 in the p53 gene in human cells, [13, 14]. Since inhaled acrolein is ultimately excreted into urine and accumulates in the bladder (*14, 15*), we hypothesize that acrolein in the urine causes DNA damage, inhibits DNA repair, and induces mutations in the urothelial cells, thereby initiating bladder carcinogenesis.

To begin to test this hypothesis we have analyzed the levels of acrolein-dG DNA adducts versus 4-ABP-DNA adducts in normal human urothelial mucosa, as well as in bladder tumor tissues. We found that the acrolein-dG DNA adduct levels in normal human urothelial mucosa and bladder tumor tissues are 10-30 fold higher than the levels of 4-ABP-DNA adducts, and that acrolein-dG DNA adduct levels in bladder tumor tissues are significantly higher than in normal human urothelial mucosa. Furthermore, we found that while both acrolein-dG DNA adducts and 4-ABP-DNA adducts are mutagenic, the former are more mutagenic and that the mutational spectra induced by these two types of DNA adducts are different. We also found that acrolein inhibits both nucleotide excision repair and base excision repair and induces repair protein degradation in human urothelial cells. Based on these results, we propose that acrolein is a major human bladder carcinogenic agent in tobacco smoke and that the susceptibility to tobacco smoke-induced acrolein-dG DNA adduct formation is a crucial factor for bladder cancer susceptibility.

## RESULTS

### Acrolein-dG adduct analysis in normal human urothelial mucosa and bladder tumor tissue genomic DNA

**Figure 1:**
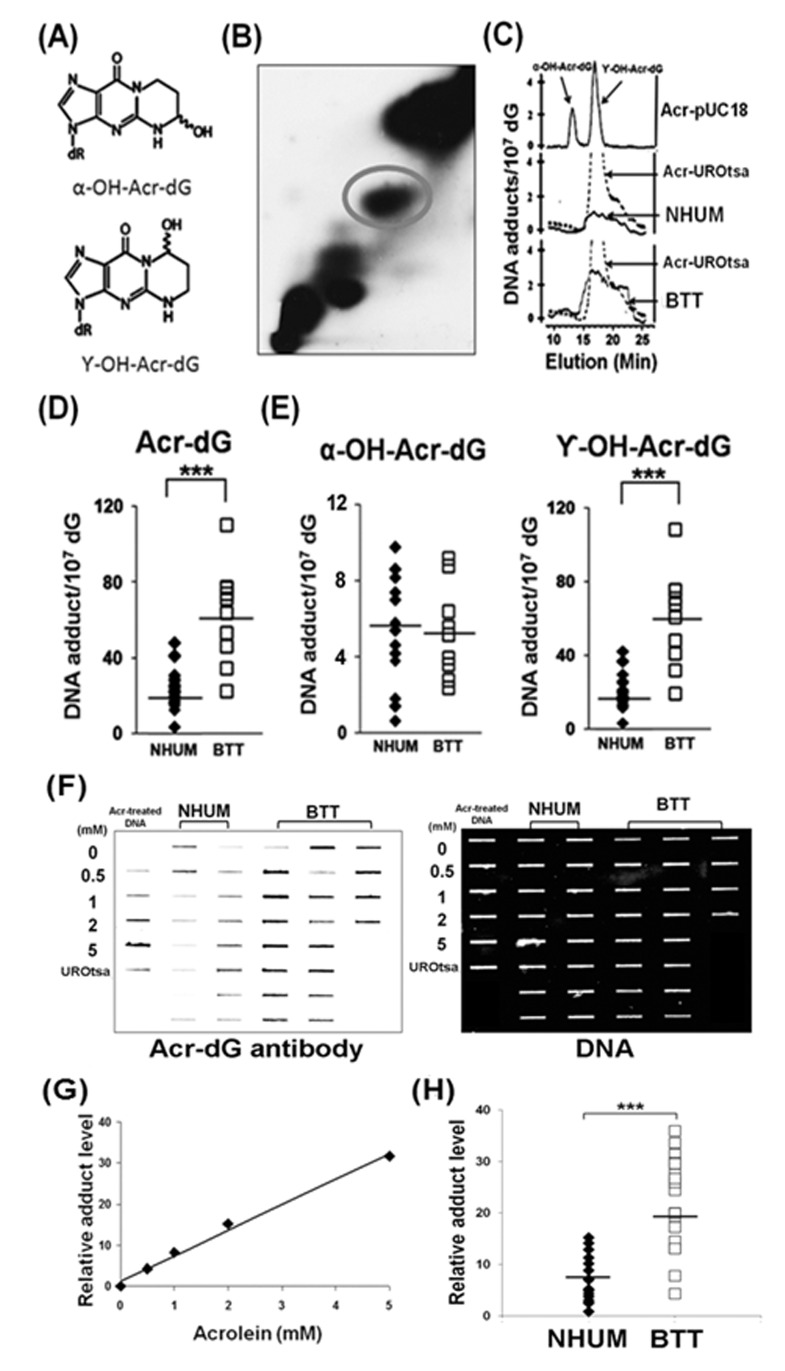
Acrolein (Acr)-dG DNA adduct analysis in normal human urothelial mucosa (NHUM) and bladder tumor tissue (BTT) samples (A) Chemical structures of α-OH-acrolein-dG and γ-OH-acrolein-dG. Genomic DNA from normal human urothelial mucosa and bladder tumor tissues were prepared and the acrolein-dG DNA adduct levels were determined by both a ^32^P-postlabeling two dimensional TLC/HPLC method (B to E) and by an immunochemical method (F to H) as described (*13, 14*). (B) A typical two dimensional TLC separation profile of isomeric acrolein-dG DNA adducts formed in normal human urothelial mucosa. The acrolein-dG DNA adducts spots (circled) resolved by two dimensional TLC (B) were extracted and further separated by HPLC (C). Similar results were observed for bladder tumor tissues. (C) The HPLC profiles of DNA adducts from acrolein-modified plasmid pUC18, acrolein-treated UROtsa cells, normal human urothelial mucosa, and bladder tumor tissues were compared. (D & E) Levels of total acrolein-dG (α-OH-acrolein-dG and γ-OH-acrolein-dG adducts) in normal human urothelial mucosa [mean ± s.d. = (25±10) X10^−7^/dG, n=19] and in bladder tumor tissues [mean ± s.d. = (63 ± 25) × 10^−7^/dG, n=10. Bars represent the mean value; statistical significance was analyzed with Student T-test. *** represents p value < 0.001. Genomic DNA from normal human urothelial mucosa (NHUM) (NB-2, NB-17 to NB-74, n=16) and bladder tumor tissue (BTT) (BT-1 to BT-75, n=20) used for acrolein-dG DNA adduct analysis by the ^32^P-postlabeling and two dimensional TLC/HPLC method were used for acrolein-dG DNA adduct detection by an acrolein-dG primary antibody and a quantum dot labeled second antibody in a slot blot apparatus as described [14]. (F) A typical slot blot result is shown. DNA was spotted on the membrane, hybridized with the acrolein-dG antibody, and then the quantum dot conjugated second antibody: first lane, plasmid pUC18 DNA modified with different concentrations of acrolein and DNA isolated from cultured UROtsa cells; second and third lanes, DNA from normal human urothelial mucosa samples; fourth, fifth and sixth lanes, DNA from bladder tumor tissue samples. Left panel, fluorescent development; right panel, the same amounts of DNA loaded in the membrane were stained with methylene blue. (G) Standard calibration curve as determined by fluorescence intensity of relative acrolein-dG DNA adduct level in plasmid pUC18 DNA modified with different concentrations of acrolein. (H) Relative acrolein-dG DNA adduct levels in normal human urothelial mucosa (n=16) and bladder tumor tissue (n=20) samples as detected by the immunochemical method as described above. Note: acrolein-dG DNA adduct levels in 10 BTT samples (BT-1 to BT-33) were detected by both ^32^P-postlabeling and the immunochemical methods. Because of the low amount of sample, 10 BTT samples (BT-54 to BT-75) were detected by immunochemical method only.

Acrolein-dG levels in the genomic DNA of normal human urothelial mucosa and bladder tumor tissues were detected by two methods: a ^32^P-postlabeling method using a two dimensional-TLC/HPLC and an immunochemical method using a monoclonal antibody specifically against acrolein-dG DNA adducts as the first antibody and a quantum dot-labeled secondary antibody [14]. For the ^32^P-postlabeling method, genomic DNA of normal human urothelial mucosa and bladder tumor tissue was isolated and digested with phosphodiesterase II and nuclease P1 to single nucleotide levels; the nucleotides, including the adducted nucleotides, were 5'-end labeled with ^32^P by T4 kinase and then subjected to two dimensional-TLC and HPLC separation as previously described [13]. Figures 1 B & 1C shows typical two dimensional-TLC and HPLC separation results of acrolein-dG DNA adducts from normal human urothelial mucosa and bladder tumor tissue samples. The results shows that γ-OH-acrolein-dG is the major isomer and α-OH-acrolein-dG is the minor isomer in both normal human urothelial mucosa and bladder tumor tissue, which is similar to the acrolein-dG DNA adducts detected in acrolein-treated cultured human cells [25, 38, 39] (Figures 1A & 1C). The levels of acrolein-dG DNA adducts in a total of 19 normal human urothelial mucosa (NB-2 to NB-74) and 10 bladder tumor tissue samples (BT-1 to BT-33) were quantified (Supplementary Table S1). The levels of acrolein-dG DNA adducts ranged from 4.0 × 10^−7^/dG to 48.2 × 10^−7^/dG [mean ~ (25±10) ×10^−7^/dG] in normal human urothelial mucosa and from 22.4 × 10^−7^/dG to 110.8 × 10^−7^/dG [mean ~ (63±25) ×10^−7^/dG] in bladder tumor tissue (Figure 1D and Supplementary Table S2). The average level of total acrolein-dG DNA adducts as well as γ-OH-acrolein-dG DNA adducts in bladder tumor tissue was 2 fold higher than in normal human urothelial mucosa while α-OH-acrolein-dG DNA adducts were not significantly different (Figures 1D & 1E).

The levels of acrolein-dG DNA adducts in the genomic DNA were also assessed immunochemically using a slot blot method, as previously described [14]; Figure 1F & 1G show typical slot blot results. The relative amounts of acrolein-dG DNA adducts in normal human urothelial mucosa (NB-2 and NB-17 to NB-74, n=16) and bladder tumor tissues (BT-1 to BT-75, n=20) were quantified based on the fluorescent intensity; the results in Figure 1H show that the average level of acrolein-dG DNA adduct in bladder tumor tissue samples was 2 fold higher than in the normal human urothelial mucosa samples, consistent with the results from the ^32^P-postlabeling method (Figure 1D).

### 4-ABP-DNA adduct analysis in normal human urothelial mucosa and bladder tumor tissue genomic DNA

**Figure 2:**
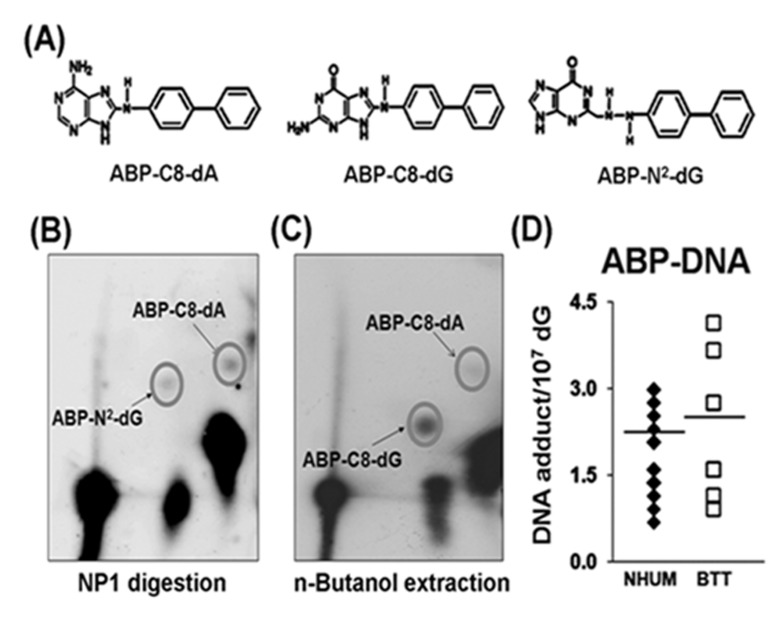
4-ABP-DNA adduct analysis in normal human urothelial mucosa (NHUM) and bladder tumor tissue (BTT) samples (A) Chemical structures of three 4-ABP-DNA adducts: 4-ABP-C8-dG, 4-ABP-N^2^-dG and 4-ABP-C8-dA. The same genomic DNA samples isolated from the normal human urothelial mucosa and bladder tumor tissues that used for acrolein-dG DNA adduct analysis were used for 4-ABP-DNA adduct analysis by the methods described in the text. (B) A typical three dimensional TLC separation of resultant nucleotides from DNA digested with nuclease P1 (NP1). This method is specifically for 4-ABP-N^2^-dG and 4-ABP-C8-dA adduct analysis. (C) A typical three dimensional TLC result from *n*-butanol extractions. This method is specifically for 4-ABP-C8-dG adduct analysis. (D) Levels of 4-ABP-DNA adducts in normal human urothelial mucosa [mean ± s.d. = (1.8±0.6) X10^−7^/dG, n=19] and bladder tumor tissue [mean ± s.d. =(2.1±1.1) × 10^−7^/dG, n=10], P = 0.32.

It is well established that 4-ABP-induces three different adducts: 4-ABP-C8-dG, 4-ABP-N^2^-dG, and 4-ABP-C8-dA (Figure 2A), which can be distinguished by differences in DNA digestion properties [32, 33]. 4-ABP-N^2^-dG and 4-ABP-C8-dA, similar to many bulky DNA adducts, are resistant to nuclease P1 digestion, and therefore, can be enriched by a nuclease P1 digestion method similar to that used for acrolein-dG DNA adduct detection and then separated by three dimensional TLC [32, 33]. 4-ABP-C8-dG adducts are sensitive to nuclease P1 digestion [32]; therefore, to analyze 4-ABP-C8-dG adducts, the nuclease P1 digestion step was omitted [16, 32]. To detect this type of adduct, genomic DNA was digested with phosphodiesterase II, the resultant nucleotides were extracted repeatedly with *n*-butanol; after evaporation of *n*-butanol, the nucleotides were dissolved in water and labeled with γ-^32^P ATP. The remaining steps were similar to those used for detecting 4-ABP-N^2^-dG and 4-ABP-C8-dA adducts [16, 40]. These adducts are separated by different solvent systems; a typical separation of the three types of 4-ABP-DNA adducts is shown in Figures 2B & 2C. The levels of 4-ABP-DNA adducts in the same normal human urothelial mucosa samples (n=19) and bladder tumor tissues (BT-1 to BT-33, n=10) used for acrolein-dG DNA adduct analyses were determined and the results shown in Figure 2D and Supplementary Table S2. 4-ABP-DNA adduct levels ranged from 0.69 × 10^−7^/dG to 2.99 × 10^−7^/dG [mean ~ (1.8±0.6) × 10-7/dG] in normal human urothelial mucosa samples and from 0.92 × 10^−7^/dG to 4.14 × 10^−7^/dG [mean ~ (2.1±1.1) x10-7/dG] in bladder tumor tissue samples. Figure 2D and Supplementary Table S2 demonstrate that neither the levels of total 4-ABP-DNA adducts nor the levels of individual 4-ABP-DNA adducts were significantly different between normal human urothelial mucosa and bladder tumor tissue.

**Figure 3:**
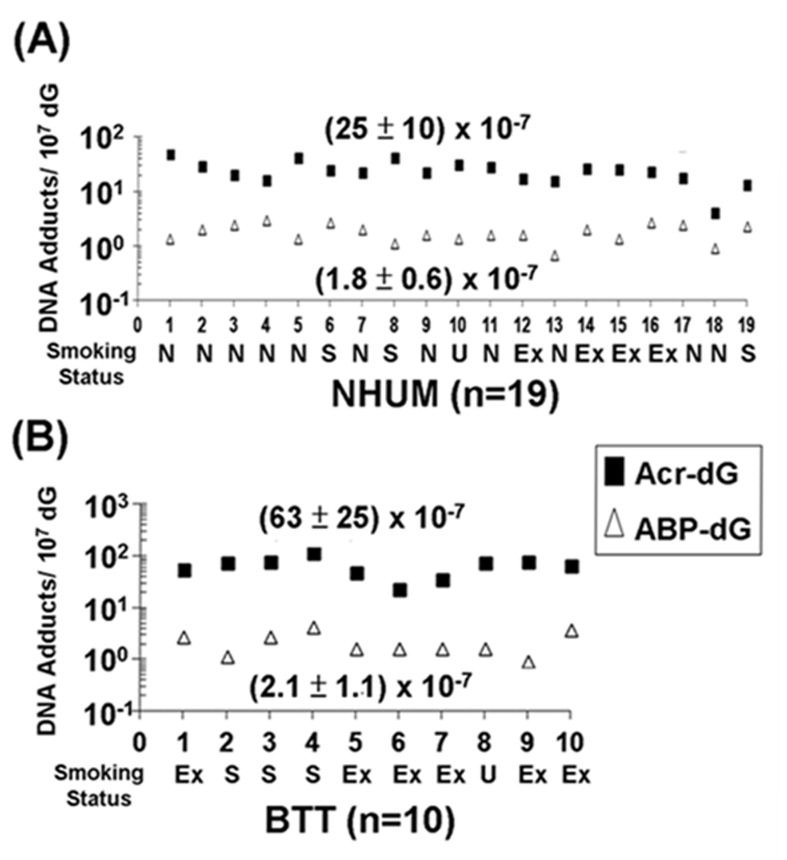
Relative levels of acrolein (Acr)-dG DNA adducts and 4-ABP-DNA adducts in normal human urothelial mucosa (NHUM) and bladder tumor tissue (BTT) samples The methods for acrolein-dG and 4-ABP-DNA adducts are described in Figs. 1 & 2. Two isomeric acrolein-dG DNA adducts and three 4-ABP-DNA adducts were quantified, as previously described [25, 32, 33]. (A) The levels of total acrolein-dG DNA adducts versus the levels of total 4-ABP-DNA adducts in normal human urothelial mucosa (n=19); and (B) the levels of total acrolein-dG DNA adducts versus the levels of total 4-ABP-DNA adducts in bladder tumor tissue (n=10). Each number along the X-axis represents a different individual (NB-2 to NB-74; BT-1 to BT-33). The tobacco smoking status of these individuals is indicated below the number with N represents non-smokers, S represents smokers, U represents unknown, and Ex represents ex-smokers. The amounts of tobacco consumption and smoking history of smokers are unknown. Note: acrolein-dG DNA adduct levels are 10-30 fold higher than 4-ABP-DNA adduct levels in normal human urothelial mucosa and bladder tumor tissues. Acrolein-dG DNA adduct levels are higher (>2 fold) in bladder tumor tissues than in normal human urothelial mucosa (p<0.001). No significant difference was observed in 4-ABP-DNA adduct levels between normal human urothelial mucosa and bladder tumor tissues (p = 0.32).

We then compared the levels of acrolein-dG DNA adducts and 4-ABP-DNA adducts in the genomic DNA of normal human urothelial mucosa (NB-2 to NB-74, n=19) and bladder tumor tissues (BT-1 to BT-33, n=10), and determined that the levels of acrolein-dG DNA adducts in normal human urothelial mucosa and bladder tumor tissue were 10-30 fold higher than the levels of 4-ABP-DNA adducts (Figures 3A & 3B).

### Mutagenicity and mutational spectrum induced by acrolein-dG DNA adducts and 4-ABP-DNA adducts in human bladder cells

**Figure 4:**
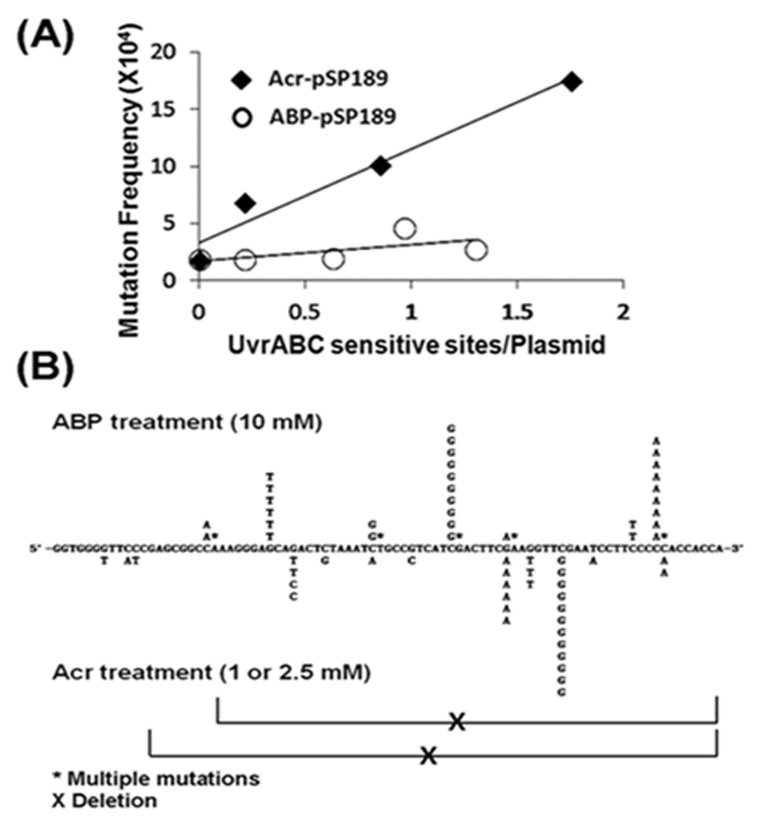
Relative mutagenicity, mutational signature and mutational spectrum induced by acrolein (Acr)-dG DNA adducts versus 4-ABP-DNA adducts in human urothelial cells The *supF* containing plasmid pSP189 DNA was either reacted with acrolein or N-OH-4-ABP and the DNA adduct number per supercoiled plasmid DNA was determined by a UvrABC incision method (Supplementary Figure S3) [25]. Modified plasmids were transfected into UROtsa cells, mutations in the *supF* gene were detected, quantified, and sequenced as previously described [25, 31]. (A) Mutation frequency per adduct induced by acrolein-dG DNA adducts and 4-ABP-DNA adducts. (B) Mutational signature and spectrum induced by acrolein-dG DNA adducts and 4-ABP-DNA adducts in the *supF* gene. Note: acrolein-dG DNA adducts are 5-6 fold more mutagenic than 4-ABP-DNA adducts. Both mutational signature (also see Table 1) and spectrum induced by acrolein-dG DNA adducts and 4-ABP-DNA adducts are different.

Although the levels of acrolein-dG DNA adducts in the normal human urothelial mucosa were much higher than the levels of 4-ABP-DNA adducts, in order to evaluate their role in bladder carcinogenesis it was necessary to measure the relative mutagenicity of these two types of DNA adducts. To achieve this goal, we reacted *supF*-containing plasmid DNA with different concentrations of acrolein and N-OH-4-ABP and determined the number of acrolein-dG DNA adducts and 4-ABP-DNA adducts by a UvrABC incision method (Supplementary Figure S3) [25]. We then determined the induction of mutations by the acrolein-dG DNA adducts and 4-ABP-DNA adducts in bladder cells using a method previously reported [25]. The results in Table 1A show that, as a function of concentration, N-OH-4-ABP is less efficient than acrolein at inducing mutations, not only in bladder cells but also in normal human lung fibroblasts. Two possibilities could account for these results: acrolein-dG DNA adducts are more mutagenic than 4-ABP-DNA adducts, and/or acrolein is more efficient than N-OH-4-ABP at inducing DNA adducts. To differentiate between these two possibilities, the number of mutations per DNA adduct induced by these two types of DNA adducts was calculated and is presented in Figure 4A. The result shows that acrolein-dG DNA adducts are 5.9-fold more mutagenic than 4-ABP-DNA adducts (p<0.05). These results are consistent with the results of Melchior et al. that show 4-ABP-DNA adducts are the least mutagenic when compared to other aromatic amines and bulky chemical-induced DNA damage [41].

Table 1Comparison of the mutation frequency (A) and types of mutations (B) in the *supF* gene in UROtsa cells and normal human lung fibroblast (NHLF) cells incubated with acrolein or N-OH-4-ABP

**(A):** 

UROtsa	mM	White colonies / Total colonies	Mutation Frequency (X10^4^)	Fold change
Acrolein	0	2/10,871	1.8	
	0.5	6/8,778	6.8	3.7
	1	9/8,856	10.1	5.5
	2.5	57/25,410	22.4	12.2
N-OH-4-ABP	0	2/10,875	1.8	
	1	2/10,591	1.9	1.0
	5	3/10,291	2.3	1.2
	10	20/42,587	4.7	2.6
	15	5/17,940	2.8	1.5
NHLF	mM			
Acrolein	0	5/29550	1.7	
	0.5	27/23328	11.6	6.8
	1	44/18094	24.3	14.4
	2	83/19084	43.5	25.7
N-OH-4-ABP	0	1/8320	1.2	
	0.5	5/9864	5.1	4.2
	5	7/9240	7.6	6.3
	10	9/9856	9.1	7.6
	15	7/9152	7.7	6.4

**(B):** 

	UROtsa	
	N-OH-4-ABP treatment	Acrolein treatment
Base substitution^a^		
G·C to T·A	15 (50%)	10 (30%)
G·C to A·T	3 (10%)	7 (20%)
G·C to C·G	12 (40%)	16 (47%)
A·T to T·A	0 (0%)	1 (3%)
A·T to C·G	0 (0%)	0 (0%)
A·T to G·C	0 (0%)	0 (0%)
Total	30	34
Single base substitution	10 (50%)	34 (81%)
Single base deletion	0 (0%)	0 (0%)
Fragment deletion	0 (0%)	8 (19%)
Multiple mutations^b^	10 (50%)	0 (0%)
Single base insertion	0 (0%)	0 (0%)
Total	20	42

a Including single base substitution and multiple mutations.

b Two mutations occur in the same plasmid.

To determine whether 4-ABP-DNA adducts and acrolein-dG DNA adducts generate different types of mutations or different mutational spectra, the *supF* mutants induced by 4-ABP (n=20, from plasmid incubated with 10 mM *N*-OH-4-ABP) and acrolein (n=42, from plasmid incubated with 1 and 2.5 mM acrolein) in UROtsa cells, were sequenced. The results in Figure 4B and Table 1B show that *supF* mutations induced by 4-ABP-DNA adducts and acrolein-dG DNA are different not only in mutational types but also have different sequence preference in the *supF* gene. Most notably, there were two –TTCGAA- mutational hotspots for acrolein modifications, while with 4-ABP, mutational hotspots occurred at a -ATCGA- site and within a run of 5 G's. The order of mutation frequency induced by ABP-DNA adducts is G to T, G to C and G to A and 4-ABP-DNA adducts induce a high frequency of multiple mutations; these results are similar to those described by Melchoir et al. [41]. In contrast, acrolein-dG adducts induce a high frequency of G to C mutations then G to T and G to A; acrolein also induces a high frequency of large deletion mutations (Table 1B and Figure 4B).

### Effect of acrolein treatment on DNA repair in human urothelial cells

**Figure 5:**
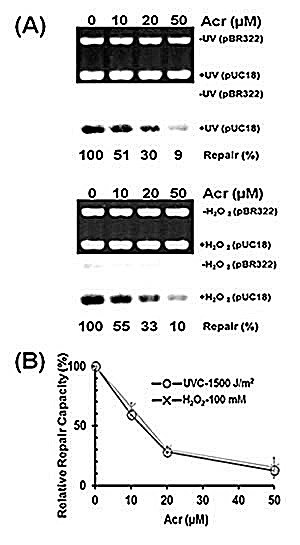
Acrolein treatment inhibits nucleotide excision repair and base excision repair in human urothelial cells Immortalized human urothelial (UROtsa) cells were treated with 0, 10, 20, or 50 μM acrolein for 1 h at 37 °C. The nucleotide excision and base excision repair capacity in these cells were determined by (A) an *in vitro* DNA damage dependent repair synthesis assay using UV-irradiated or H_2_O_2_ modified pUC18 plasmid DNA as substrates, and (B) a host cell reactivation assay using UV-irradiated or H_2_O_2_ modified luciferase plasmid pGL3 as substrates, as previously described (*13, 26*).

Previously we have found that acrolein can not only damage DNA but also modify repair proteins causing DNA repair dysfunction in normal human bronchial epithelial cells (*26*). To test whether or not acrolein can cause the same effect in human urothelial cells, we determined the effect of acrolein treatment on DNA repair capacity for both bulky DNA damage and oxidative DNA damage using a host cell reactivation assay and an *in vitro* DNA damage dependent repair synthesis assay (*13, 26*). Results in Figure 5 show that acrolein treatment causes a concentration-dependent reduction of DNA repair capacity for both UV-induced DNA damage and oxidative DNA damage. Since it is well established that UV-induced DNA damage is repair mainly by nucleotide excision repair mechanism and oxidative DNA damage is repair by base excision repair mechanism (26), these results indicate that acrolein treatment can inhibit both nucleotide excision repair and base excision repair.

**Figure 6:**
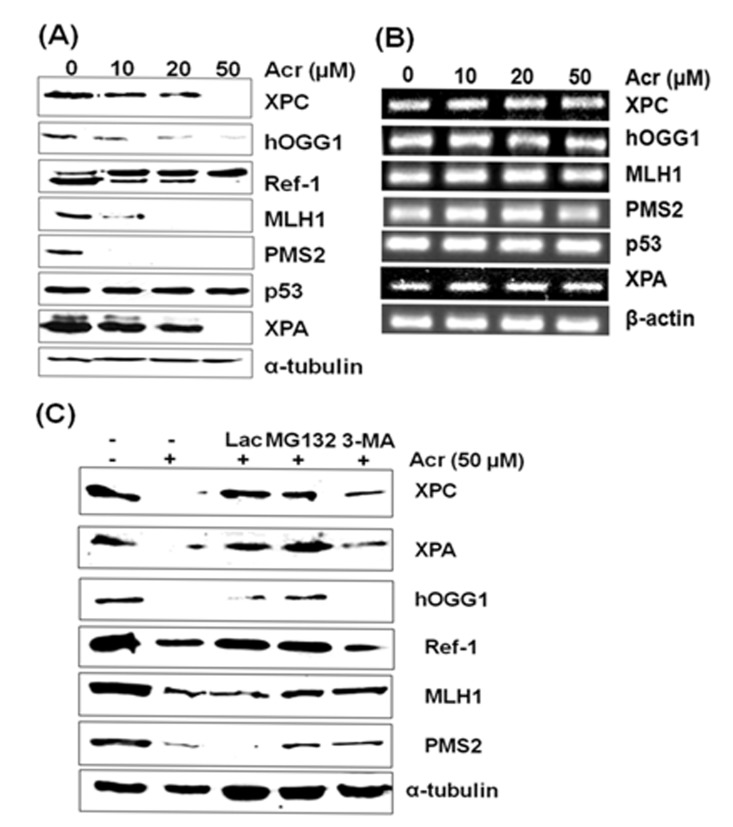
Effect of acrolein treatment on protein and mRNA levels of XPA, XPC, hOGG1, MLH1, PMS2, and Ref-1 gene products in urothelial cells UROtsa cells were treated with different concentrations of acrolein for 1 h at 37 °C, the proteins were separated by SDS-PAGE and detected by Western blot (A), and the mRNA levels were detected by RT-PCR (B). In (C) proteosome inhibitor Lactacystin (10 μM) and MG132 (20 μM) and autophagosome inhibitor 3-methyladenine (10 mM) was added to UROtsa cells before acrolein treatment and the proteins were detected. Note: 1) Acrolein treatment induces a dose dependent reduction of XPA, XPC, hOGG1, MLH1 and PMS2 proteins but not p53 and α-tubulin; 2) Proteosome inhibitor Lac and MG132 inhibits the reduction of XPA, XPC, hOGG1, Ref-1, MLH1 and PMS2 proteins induced by acrolein treatments. Autophagosome inhibitor 3-MA partially inhibits the reduction of XPA, XPC, Ref-1, MLH1 and PMS2.

There are three possible mechanisms via which that acrolein causes inhibition of DNA repair in urothelial cells: one, acrolein modifies repair protein causing protein dysfunction; two, acrolein suppresses expression of repair genes; and three, acrolein-repair protein modifications induce protein degradation. To distinguish these possibilities we determined the effect of acrolein treatment on repair protein levels as well as expression of repair genes. We found that acrolein treatment causes a dose-dependent reduction of XPA, XPC, hOGG1, PMS2, and MLH1 proteins but has no effect on p53. In contrast, acrolein treatment does not affect the mRNA levels of these genes (Fig. 6A & 6B). These results further indicate that acrolein impairs NER, and BER processes through its specific effects on repair proteins rather than their gene transcriptions. It has been long recognized that acrolein can form Schiff base and carbonylate with amino acid such as lysine, cysteine, and histidine [26, 42]. It is possible that acrolein modifications cause protein conformation change and that these acrolein-modified proteins are subjected to degradation via proteasome and/or autophagosome similar to multiple ubiquitinated proteins [43, 44]. To test this possibility, cells were pretreated with the proteasome inhibitor MG132, Lactacystin (Lac) and autophagy inhibitor 3-methyladenine (3-MA). Results in Fig. 6C show that acrolein does not cause a reduction of repair proteins in cells pretreated with the proteasome inhibitor MG132 and Lac, indicating that acrolein-modified repair proteins are degraded by proteasomes. However, 3-MA can also partially inhibits degradation of XPC, XPA, Ref-1, MLH1 and PMS2 but not hOGG1. These results suggest that acrolein-modified repair proteins are degraded via both proteosome and autophagy pathways or via proteosome pathway but that 3-MA may be able to partially inhibit this pathway.

## DISCUSSION

Bladder cancer is the most often occurring secondary cancer in patients who have undergone a prior extended regimen of cyclophosphamide and ifsofamide treatment [19, 20, 45]. Since a major metabolite of cyclophosphamide and ifsofamide in humans is acrolein, and because acrolein can induce mutagenic acrolein-dG DNA adducts and can induce bladder tumors in rat models, acrolein has long been suspected to be a human bladder carcinogen [18-20]. However, the role of acrolein in tobacco smoke-related bladder cancer has not previously been established [18, 21, 46]. Instead, arylamines, specifically 4-ABP, have been generally recognized as bladder cancer causing agents in tobacco smoke, despite the fact that the amount of arylamines in tobacco smoke is 50,000 fold less than acrolein [8, 9]. We recently found that acrolein can function as a co-mutagen, enhancing DNA damaging agent-induced mutagenesis while concurrently causing DNA repair dysfunction by modifying repair proteins [26]. Together, these results suggest that acrolein is a major bladder cancer etiological agent in tobacco smoke. In this study, we found that acrolein-dG DNA adducts were more abundant than 4-ABP-DNA adducts in normal human urothelial mucosa as well as in bladder tumor tissues and that acrolein-dG DNA adducts were more mutagenic than 4-ABP-DNA adducts. We also found that acrolein treatment caused an inhibitory effect on both nucleotide excision repair and base excision repair and repair protein degradation in human urothelial cells. These results serve as a first step in establishing the role of acrolein in human bladder cancer.

Acrolein is not only abundant in tobacco smoke, it is also abundant in automobile exhaust and cooking fumes [23]. In addition, acrolein is a byproduct of lipid peroxidation in cells under excessive oxidative stress [26, 47]. Therefore, it is possible that acrolein from both environmental and endogenous sources can account for the relatively high levels of acrolein-dG DNA adducts observed in normal human urothelial mucosa obtained from nonsmokers. We were unable to determine the effect of tobacco smoke on acrolein-dG and 4-ABP-DNA adduct formation in urothelial mucosa due to small sample size and lack of information on individual's smoking history and amount of tobacco consumption. However, since acrolein is readily absorbed by human cells, we propose that smokers have higher levels of acrolein-dG DNA adducts in urothelial cells than nonsmokers and that the susceptibility to acrolein-dG DNA adduct formation is an important factor in determining bladder cancer susceptibility [13, 26, 48].

Acrolein is cytotoxic and can efficiently deplete antioxidants such as glutathione in lung cells [49]. It has been found that the distribution of acrolein-induced DNA damage in the p53 gene in normal human bronchial epithelial cells coincides with the lung cancer p53 mutational spectrum. Therefore, it has been proposed that acrolein is a major lung cancer causing agent in tobacco smoke and cooking fumes [13, 23]. The types of human cancer induced by acrolein appear to be limited to bladder and lung. Tobacco smoke, in contrast, induces a wide range of human cancers [6, 48, 50]. In rat models, intraperitoneal injection of acrolein with non-carcinogenic uracil specifically causes bladder tumors [18]. Acrolein can directly react with proteins and DNA to form adducts, therefore, unlike 4-ABP, the tissue specificity of acrolein carcinogenicity probably is unlikely due to expression of phase 1 detoxification enzymes in bladder and lung tissues. Rather, it is probably due to limitations of bioavailability because once acrolein is in the blood stream it readily forms conjugates with proteins and is less active and not readily be absorbed by human cells. However, protein conjugated acrolein can be reverted to free acrolein in the renal system and acrolein is excreted and therefore accumulates in urine in the bladder (*14, 15*). This may be the reason why bladder, aside from lung, which is the first major target of inhaled acrolein, is the other major target organ for acrolein-induced carcinogenesis to occur.

In animal models, the oral administration of 4-ABP induces liver and bladder cancer while subcutaneous injection can cause mammary and intestine tumors [51]. It has been proposed that arylamines are metabolized to *N*-hydroxyamines in the liver and transported to the bladder as glucuronides, which are converted back to *N*-hydroxyarylamides that can be taken up by bladder epithelial cells that have abundant *N*-acetyltransferase 1 capable of converting *N*-hydroxyarylamine to a more reactive *N*-acetoxyarylamine [52, 53]. Consequently, *N*-acetoxyarylamines react with DNA to form mutagenic DNA adducts [52, 53]. These findings provide a plausible explanation of 4-ABP bladder carcinogenicity; however, the low mutagenicity of 4-ABP-DNA adducts is puzzling. Arylamine DNA adducts such as 2-aminofluorene-C8-dG DNA adducts have been found to be able to maintain *trans-anti* stereo structures that enable adducted deoxyguanosine to pair with deoxycytosine [54, 55]. Perhaps, 4-ABP-dG and 4-ABP-dA adducts have similar stereostructures as aminofluorene-C8-dG, therefore, allow efficient translesion synthesis with high fidelity [56, 57].

While the majority of literatures report that both γ-OH-acrolein-dG and α-OH-acrolein-dG adducts are mutagenic in mammalian cells [25, 48, 58-61], two reports found that γ-OH-acrolein-dG adduct is not mutagenic at a specific sequence and at a very low adduct concentrations [62, 63]. An in depth review to address the reparability and mutagenicity of these two types of adducts can be found in a recent review article [48]. It is worth noting that most recent results from Llyod's laboratory has unambiguous demonstrated that γ-OH-acrolein-dG adduct is mutagenic [59-61], and recent results from Tang's laboratory indicate that the reparability and mutagenicity of these two kinds of adducts appear to be cell type dependent [48].

We do not yet know why bladder tumor tissues have higher levels of γ-OH-acrolein-dG DNA adducts than those in normal human urothelial mucosa. It is possible that tumor cells are more susceptible to γ-OH-acrolein-induced DNA adduct formation and/or tumor cells have lower repair capacity for this type of DNA adducts. It should be noted that *in vitro* acrolein-DNA modifications generate mainly γ-OH-acrolein-dG adducts under neutral or slightly alkaline conditions (pH < 8.0), and only under high pH conditions (pH 10) do α-OH-acrolein-dG adducts become significant (26, 44). Acrolein is a byproduct of lipid peroxidation, which can be triggered by oxidative stress [48]. Perhaps, the higher acrolein levels in the bladder tumor cells than in normal human urothelial mucosa are due to higher levels of oxidative stress in tumor cells, which consequently leads to the formation of higher levels of acrolein-dG DNA adducts.

In summary, we found that the levels of acrolein-dG DNA adducts are 10-30 fold higher than the levels of 4-ABP-DNA adducts in both normal human urothelial mucosa and bladder tumor tissues, that acrolein inhibits nucleotide excision repair base excision repair, and that acrolein-dG DNA adducts are much more mutagenic than 4-ABP-DNA adducts. Since tobacco smoke contains 50,000 fold more acrolein than arylamines, these results strongly suggest that acrolein may play a more important role than 4-ABP in tobacco smoke-related bladder carcinogenesis.

## MATERIALS AND METHODS

### Materials

Uvr proteins were purified as previously described [27]. Monoclonal antibodies to acrolein-dG DNA adducts were prepared as described [14]. UPIII antibodies were purchased from Abcam (Cambridge, MA), α-tubulin from Calbiochem (Billerica, MA), and antibodies against mouse/rabbit IgG from Amersham Biosciences (Pittsburgh, PA). Normal human bladder epithelial cells (HBEP cells) were obtained from Zen-Bio (Research Triangle Park, NC). UROtsa cells were obtained from the laboratory of JR Masters (University College London, UK) and normal human fibroblasts (CCL202 cells) were obtained from ATCC (American Type Culture Collection, Manassas, VA). Plasmid pSP189 was prepared as described by Canella and Seidman [28]. T4 kinase, protease K, and RNase A were purchased from New England Biolabs (Ipswich, MA); γ-^32^P-ATP and α-^32^P-dATP were purchased from Perkin Elmer (Waltham, MA), and nuclease P1 and phosphodiesterase II were purchased from Sigma (St. Louis, MO) and Worthington (Lakewood, NJ), respectively. Acrolein (purity 90%) was purchased from Sigma and N-hydroxy-4-ABP (N-OH-4-ABP) was synthesized as previously described (34).

### Cells and Cell Cultures

Normal human bladder epithelial cells (HBEP cells) were grown in medium provided by Zen-Bio. Immortalized normal human bladder cells (UROtsa cells) were grown in serum-free media containing 1:1 mixture of Dulbecco's modified Eagle's medium [29] and Ham's F-12 supplemented with selenium (5 ng/ml), hydrocortisone (36 ng/ml), transferrin (5 μg/ml), insulin (5 μg/ml), triiodothyronine (4 pg/ml), and epidermal growth factor (10 ng/ml). Normal human lung fibroblasts (CCL-202 cells) were grown in modified Eagle's medium [25] supplemented with 10% fetal bovine serum.

### Genomic DNA Isolation

Genomic DNA was isolated as previously described [30]. In brief, cells were washed with phosphate-buffered saline (PBS) (137 mM NaCl, 2.7 mM KCl, 10 mM Na_2_HPO_4_, 1.8 mM KH_2_PO_4_, pH7.5) and lysed with lysis buffer (0.5% sodium dodecyl sulfate, 10 mM Tris, pH 7.8, 10 mM EDTA, 10 mM NaCl, 100 μg/mL proteinase K) at room temperature for 4 h. Protein was removed by repeated phenol/diethyl ether extractions, and the DNA was precipitated with sodium acetate (0.3 M, pH 7.0) and 75% ethanol, and resuspended in TE buffer (10 mM Tris, pH 7.5, 1 mM EDTA). RNA was removed by treatment with RNase A (50 μg/mL) at 37°C for 2 h, followed by repeated phenol/diethyl ether extractions, and the DNA was precipitated by ethanol–sodium acetate and resuspended in TE buffer. The purity of DNA was assessed by gel electrophoresis and UV absorption spectral analysis.

### UPIII detection

Cell lysates from cultured bladder cells and human bladder tissues were prepared as previously described [13]. The protein levels were determined by the Bradford procedure (Bio-Rad, Hercules, CA). Western blots were performed as indicated in previous protocols [26]. Antibodies against human UPIII and α-tubulin were used as primary antibodies and horseradish peroxidase-labeled antibodies against mouse/rabbit IgG were used as secondary antibodies. The signals were developed with an enhanced chemiluminescence technique (Amersham Biosciences).

### Modifications of plasmid DNA with acrolein and N-OH-4-ABP

Plasmid DNAs (pUC18 and pSP189) were modified with 0, 0.5, 1.0, or 2.5 mM acrolein or 0, 1, 5, 10, or 15 mM N-OH-4-ABP at the 37°C for 16 h. Un-reacted acrolein and degradation products of N-OH-4-ABP were removed by repeat phenol/diethyl ether extractions, and the DNAs were precipitated and washed with ethanol and dissolved in TE buffer.

### SupF mutagenesis assay

Shuttle vector pSP189 plasmids, which contain a tyrosine suppressor tRNA coding gene *supF*, were used as mutational targets [31]. The number of DNA adducts formed in the plasmid DNA was determined by UvrABC incision method as previously described [25]. Exponentially growing UROtsa cells or normal human lung fibroblasts (CCL-202 cells) were transfected with acrolein- or N-OH-4-ABP-modified pSP189 plasmids (20 μg) using FuGENE-6 (Roche, Nutley, NJ) according to manufacturer's instructions. The plasmids were then rescued from the human cells by an alkaline lysis method 72 h after transfection [31]. Mutations were identified, and mutation frequency calculations and mutated sequence identifications in the *supF* gene were conducted as described previously [25].

### Effect of acrolein treatment on DNA repair capacity

The effect of acrolein treatment on DNA repair capacity was assessed by an *in vitro* DNA damage dependent repair synthesis assay and a host cell reactivation assay as previously described (13, 26). UROtsa cells were treated with 0, 10, 20 and 50 μM acrolein for 1 h at 37 °C. Cell lysates were prepared immediately after acrolein treatment and frozen at −80 °C. The cell lysates were used to carry out DNA damage dependent repair synthesis assay as previously described (13, 26). In brief, UV-irradiated pUC18 plasmids were as substrates for nucleotide excision repair, H_2_O_2_ modified pUC18 as substrates for base excision repair, and unmodified pBR322 as the control; DNA damage dependent repair synthesis was conducted in the presence of α-^32^P-dATP. The amount of ^32^P incorporation in the synthesized DNA after subtraction of control and normalization with the amount of input DNA represents the repair capacity (13, 26). For the host cell reactivation assay, UROtsa cells with and without acrolein treatment were transfected with UV-irradiated or H_2_O_2_-modified luciferase plasmid pGL3 and unmodified pSV-β-galactosidase plasmid; after 24 h incubations the luciferase and β-galactosidase activity in these cells were measured as previously described (13, 26). The relative luciferase after normalized with β-galactosidase represents the relative repair capacity.

### Acrolein- and 4-ABP-DNA adduct determinations

Acrolein-dG DNA adduct levels in the genome were detected by a two dimensional thin layer chromatography (TLC)/HPLC method, as well as by an immunochemical method as previously described [14, 26]. Because 4-ABP-C8-dG DNA adducts are sensitive to nuclease P1 digestion, these adducts were detected directly in *n*-butanol extracts without nuclease P1 treatment by a three dimensional TLC method [16, 32]. 4-ABP-C8-dA and 4-ABP-N^2^-dG DNA adducts were detected by nuclease P1 digestion and a three dimensional TLC method [33]. Using dGMP for a ^32^P labeling standard, we determined that the ^32^P postlabeling two dimensional TLC/HPLC method detected 5% of acrolein-dG adducts that were detected by LC-MS/MS (25) and that ^32^P postlabeling three dimensional TLC method detected 4.3% of 4-ABP-DNA adducts that were detected by LC-MS/MS [34]. The final levels of acrolein-dG and 4-ABP-DNA adducts were normalized based on these results.

### Reverse transcription-polymerase chain reaction (RT-PCR) and repair protein detections

Total RNAs were extracted using the PureLink™ RNA Mini Kit (Invitrogen). Reverse transcription was performed using the SuperScript™ III First-Strand Synthesis System (Invitrogen). The levels of repair proteins, XPA, XPC, hOGG1, Ref1, p53, MLH1 and PMS2 were detected by Western blotting as previously described [26].

### Normal human urothelial mucosa and bladder tumor tissue samples

Normal human urothelial cells were obtained by excisional biopsy of normal urothelial mucosa from the neck of the bladder undergoing radical prostatectomy (Supplementary Table S1). Based on light microscope inspection, we estimated that >95% of the samples were normal urothelial epithelial cells. Bladder tumors of various grades were diagnosed based on tumor morphology and corroborated using results from histological light microscopic examination (Supplementary Table S1). Following surgical resection, tissue samples were immediately frozen at −80 °C. To ensure that the normal human urothelium mucosal samples had the characteristics of urothelial cells, we assessed the expression of uroplakin IIIa (UPIIIa) [35, 36]. The results in Supplementary Figure S2 show that UPIIIa (molecular weight 47 kDa) is highly expressed in normal urothelial tissue samples and that the expression of UPIIIa is significantly reduced in bladder tumor tissue. A positive reaction for UPIIIa has been reported in transitional cell carcinomas; however, none of a large series of carcinomas derived from other organs that comprised 106 primary and 71 metastatic carcinomas was found to be positive for UPIIIa [37].

The study was approved by the New York University IRB. All patients provided informed consent in their native language to participate in the study.

## SUPPLEMENTARY MATERIAL TABLES AND FIGURES


