# Supplementary material for: Acrolein- and 4-Aminobiphenyl-DNA adducts in human bladder mucosa and tumor tissue and their mutagenicity in human urothelial cells
Source: Oncotarget. 2014 May 7;5(11):3526–40. doi: 10.18632/oncotarget.1954 (PMC4116500; doi:10.18632/oncotarget.1954)
Supplement: Supplementary file 1 [file oncotarget-05-3526-s001.pdf]

Acrolein- and 4-Aminobiphenyl-DNA Adducts in Human Bladder Mucosa and Tumor Tissue and Their Mutagenicity in Human Urothelial Cells

Supplementary Material

Supplementary Table S1: (A) Information on age and sex of individuals from whom the normal human urothelial mucosa were used. (B) Information on age, and sex of patients (M: male, F: female) and the grade of their bladder tumor diagnosed.
*N: non-smoker, S: smoker, U: smoking history unknown, Ex: ex-smoker.

(A) Normal human urothelial mucosa				(B) Bladder tumor tissues					
Patient	Age	Sex	Smoking history*	Patient	Age	Sex	Tumor Grade	Stage	Smoking history*
NB-2	66	M	N	BT-1	73	M	Low	Ta	Ex
NB-7	68	M	N	BT-3	66	M	High	T1	S
NB-15	65	M	N	BT-4	63	M	High	T1	S
NB-16	65	M	N	BT-8	73	M	High	Ta	S
NB-17	64	M	N	BT-10	56	M	High	T3/T4	Ex
NB-22	62	M	S	BT-26	84	M	High	T1	Ex
NB-23	53	M	N	BT-30	84	M	High	T1	Ex
NB-25	64	M	S	BT-31	68	F	High	T2	U
NB-28	65	M	N	BT-32	54	M	High	T2	Ex
NB-57	63	M	U	BT-33	57	F	Low	Ta	Ex
NB-58	79	M	N	BT-54	68	F	High	Ta	Ex
NB-65	57	M	Ex	BT-55	65	M	High	T1	S
NB-66	71	M	N	BT-56	73	F	High	T1	Ex
NB-67	69	M	Ex	BT-61	98	M	High	T2	N
NB-68	60	M	Ex	BT-62	54	M	High	T3/T4	N
NB-69	67	M	Ex	BT-63	77	M	High	T3/T4	Ex
NB-70	75	M	N	BT-64	79	M	High	T2	U
NB-71	56	M	N	BT-72	71	M	High	T2	N
NB-74	58	M	S	BT-73	78	M	High	T2	Ex
				BT-75	58	M	High	T2	S

Supplementary Table S2: The levels of α-OH-acrolein-dG, γ-OH-acrolein-dG, 4-ABP-C8-dG, 4-ABP-N^2-dG and 4-ABP-C8-dA in normal human urothelial mucosa (NB, n=19) and bladder tumor tissues (BT, n=10).

DNA adduct/ dG x 10^7							
	Acrolein-dG adducts			ABP-DNA adducts			
Tissue	α-OH-acrolein-dG	ϒ-OH-acrolein-dG	Total	4-ABP-N^2-dG	4-ABP-C8-dG	4-ABP-C8-dA	Total
NB-2	10.00	38.20	48.20	0.23	0.23	0.92	1.38
NB-7	4.80	24.40	29.20	0.23	0.69	1.15	2.07
NB-15	4.40	15.80	20.20	0.46	0.92	1.15	2.53
NB-16	5.60	10.60	16.20	0.69	0.92	1.38	2.99
NB-17	7.20	33.80	41.00	0.23	0.46	0.69	1.38
NB-22	8.40	16.00	24.40	1.15	0.46	1.15	2.76
NB-23	8.80	13.60	22.40	0.46	0.46	1.15	2.07
NB-25	7.60	34.20	41.80	0.23	0.46	0.46	1.15
NB-28	8.80	13.40	22.20	0.23	0.46	0.92	1.61
NB-57	1.60	29.60	31.20	0.23	0.46	0.69	1.38
NB-58	4.80	23.20	28.00	0.23	0.46	0.92	1.61
NB-65	2.00	15.20	17.20	0.23	0.46	0.92	1.61
NB-66	0.80	14.80	15.60	0.23	0.23	0.23	0.69
NB-67	8.40	17.80	26.20	0.23	0.69	1.15	2.07
NB-68	8.80	16.60	25.40	0.23	0.69	0.46	1.38
NB-69	6.00	16.80	22.80	0.46	0.69	1.61	2.76
NB-70	4.00	14.00	18.00	0.46	1.15	0.92	2.53
NB-71	0.80	3.20	4.00	0.23	0.23	0.46	0.92
NB-74	2.00	11.00	13.00	0.69	0.46	1.15	2.30
BT-1	8.80	44.6	53.4	0.69	0.69	1.38	2.76
BT-3	6.40	65.2	71.6	0.46	0.23	0.46	1.15
BT-4	2.40	75.2	77.6	0.46	0.92	1.38	2.76
BT-8	3.60	107.2	110.8	2.76	0.46	0.92	4.14
BT-10	9.20	37	46.2	0.46	0.69	0.46	1.61
BT-26	5.20	17.2	22.4	0.46	0.46	0.69	1.61
BT-30	4.00	30.8	34.8	0.69	0.46	0.46	1.61
BT-31	5.20	69.2	74.4	0.46	0.46	0.69	1.61
BT-32	2.80	74.6	77.4	0.23	0.23	0.46	0.92
BT-33	5.60	58.6	64.2	0.69	0.46	2.53	3.68

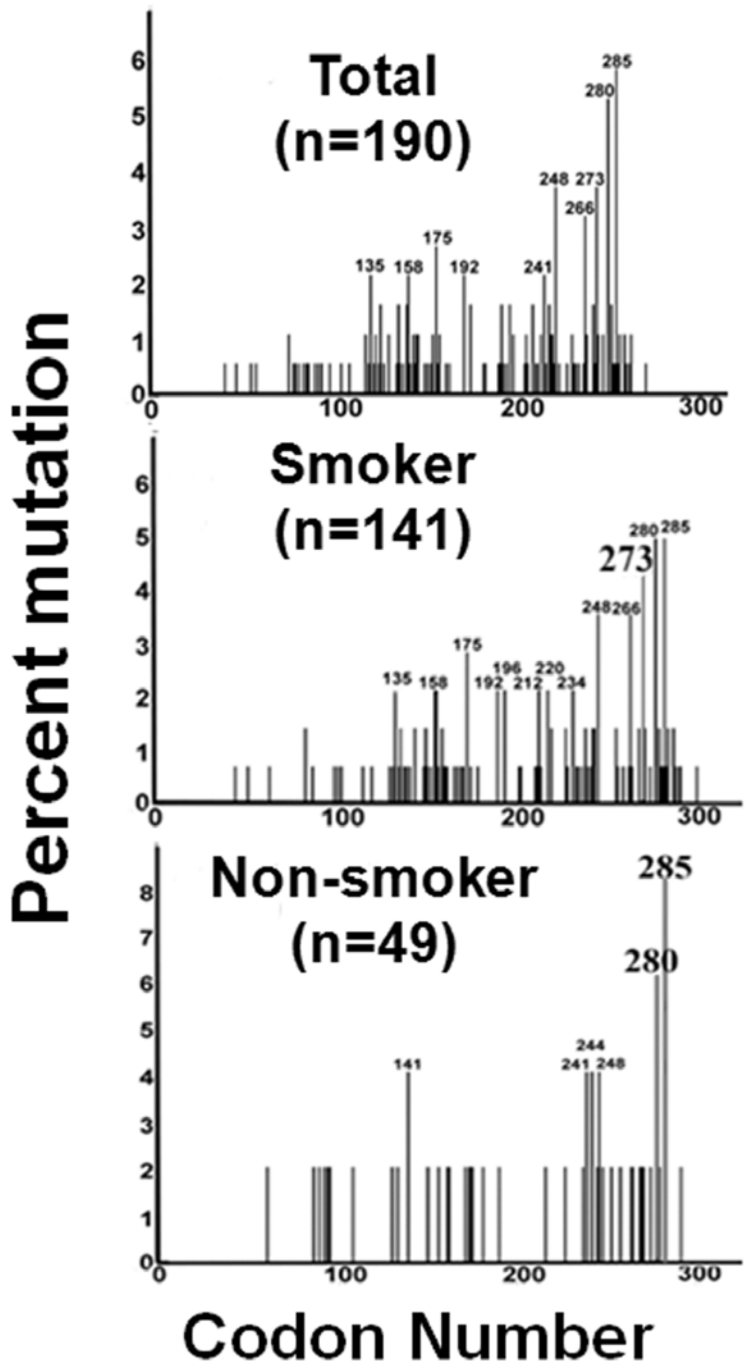

Supplementary Figure S1: Mutation (base substitutions) distribution in the p53 gene in bladder cancer. Mutations in the p53 gene in bladder cancer (n=190) were identified by DNA sequencing. The results were obtained from the International Agency for Research on Cancer data base, http:www.p53.iarc.fr [12]. The smoking histories of 190 entries were identified and the p53 codon mutation distributions in non-smokers and smokers are shown. Note: codon 273 is a mutational hotspot for bladder cancer of smokers only and codons 280 and 285 for bladder cancer of both smokers and nonsmokers.

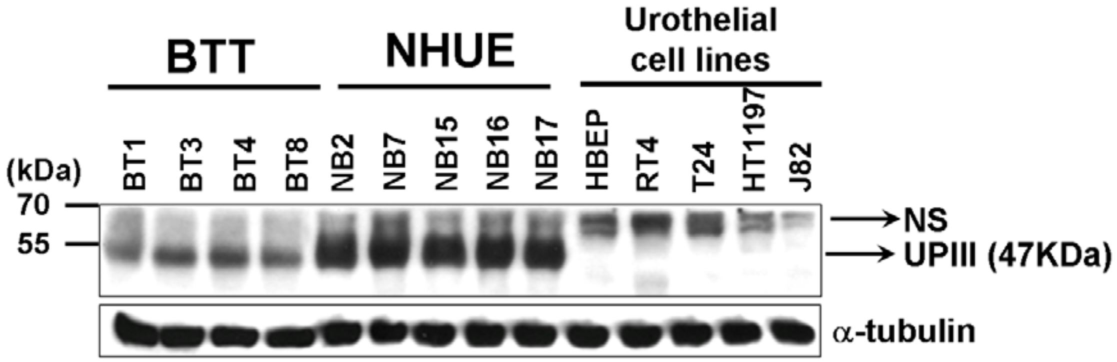

Supplementary Figure S2: Expression of UPIII in normal human urothelial mucosa samples (NHUM), bladder tumor tissues (BTT) and cultured human urothelial cells (HBEP: human urothelial progenitor cells; RT4, T24, HT1197, and J82: bladder cancer cells). Proteins in the freshly prepared cell lysates from different samples were separated by electrophoresis and UPIII and α-tubulin levels were detected by Western blot [23]. Note: UPIII (47 kDa) is highly expressed in NHUM *in vivo*, reduced in BTT, but not in cultured human urothelial cells. NS: non-specific bands. UPIII expression was checked in all normal human urothelial mucosa samples (n=19), and bladder tumor tissues (n=20) used for this study.

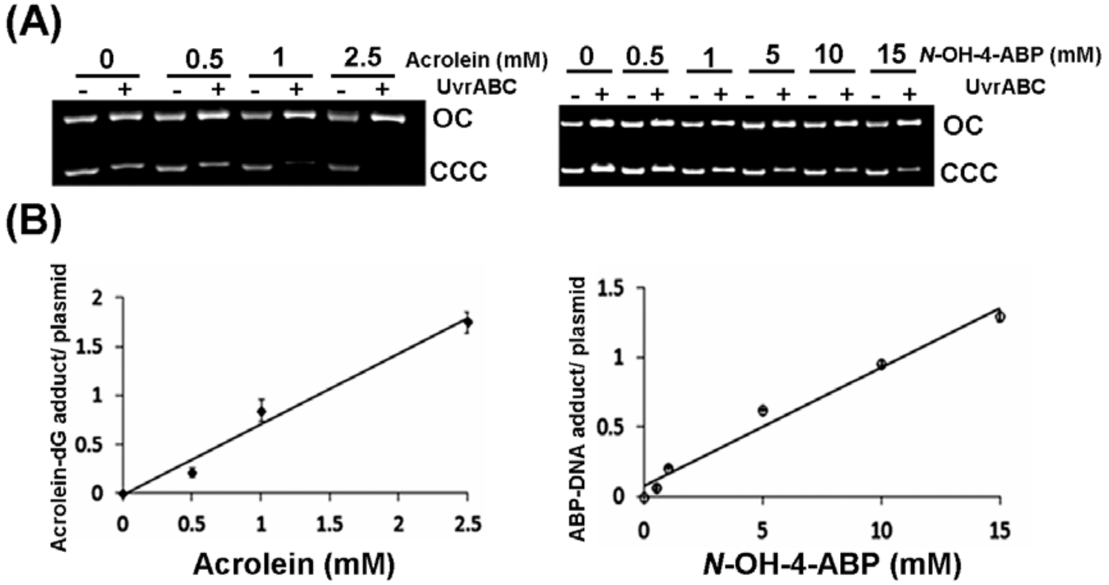

Supplementary Figure S3: Acrolein-dG DNA adducts and 4-ABP-DNA adducts as detected by an UvrABC incision method. Supercoiled pSP189 DNA modified with different concentrations of acrolein and *N*-OH-4-ABP was reacted with UvrABC and the resultant DNA was separated by electrophoresis and number of UvrABC incisions, which represent the number of DNA adducts, was quantified by the Poisson distribution equation the same as previously described [22]. (A) Typical gel electrophoresis results, and (B) quantification of adduct number per plasmid DNA molecule. OC, open circle; CCC, covalently closed circle. Note: The sensitivity of UvrABC detection for acrolein-dG adducts is comparable to LC-MS/MS method [22], and it has been shown that UvrABC method can detect ABP-DNA adduct quantitatively and specifically [10, 11], therefore, this method is the choice for quantifications of these two types of DNA adducts formed in the pSP189 plasmid DNA.
